# Crystal structure and Hirshfeld surface analysis of (*E*)-3-[(4-methyl­benzyl­idene)amino]-5-phenylthiazolidin-2-iminium bromide *N*,*N*-di­methyl­formamide monosolvate

**DOI:** 10.1107/S2056989020012712

**Published:** 2020-09-30

**Authors:** Gulnara Sh. Duruskari, Ali N. Khalilov, Gunay Z. Mammadova, Sevim Türktekin Çelikesir, Mehmet Akkurt, Anzurat A. Akobirshoeva, Abel M. Maharramov

**Affiliations:** a Organic Chemistry Department, Baku State University, Z. Khalilov str. 23, Az, 1148 Baku, Azerbaijan; b Department of Physics and Chemistry, "Composite Materials" Scientific Research Center, Azerbaijan State Economic University (UNEC), H. Aliyev str. 135, Az 1063, Baku, Azerbaijan; c Department of Physics, Faculty of Sciences, Erciyes University, 38039 Kayseri, Turkey; d Academy of Science of the Republic of Tadzhikistan, Kh. Yu. Yusufbekov Pamir Biology Institute, 1 Kholdorova St, Khorog 736002, Gbao, Tajikistan

**Keywords:** crystal structure, thia­zolidine, envelope conformation, *N*,*N*-di­methyl­formamide, Hirshfeld surface analysis

## Abstract

In the cation of the title salt, C_17_H_18_N_3_S^+^·Br^−^·C_3_H_7_NO, the central thia­zolidine ring adopts an envelope conformation with puckering parameters *Q*(2) = 0.310 (3) Å and *φ*(2) = 42.2 (6)°. In the crystal, each cation is connected to two anions by N—H⋯ Br hydrogen bonds, forming an *R*_4_^2^(8) motif parallel to the (10) plane. van der Waals inter­actions between the cations, anions and *N*,*N*-di­methyl­formamide mol­ecules further stabilize the crystal structure in three dimensions. The most important contributions to the surface contacts are from H⋯H (55.6%), C⋯H/H⋯C (17.9%) and Br⋯H/H⋯Br (7.0%) inter­actions, as concluded from a Hirshfeld analysis.

## Abstract 2

In the crystal, each cation is connected to two anions by N—H⋯ Br hydrogen bonds, forming an 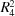
(8) motif parallel to the (10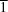) plane, while the *N*,*N*-di­methyl­formamide mol­ecules are linked to the cations by C—H⋯O contacts.

## Chemical context   

Sulfur and nitro­gen-containing heterocyclic systems are of great inter­ests in the fields of organic synthesis, drug design and material science (Abdelhamid *et al.*, 2014 ▸; Pathania *et al.*, 2019 ▸; Yin *et al.*, 2020 ▸). In this context, thia­zolidine derivatives play an important role in pharmaceutical and medicinal chemistry. Many commercially available drugs such as pioglitazone (an anti­diabetic), penicillin, benzyl­penicillin, ampicillin, oxacillin and amoxicillin (β-lactam anti­biotics) contain a thia­zolidine moiety. Studies in the field of thia­zolidine chemistry have been well documented in the literature (D’hooghe & De Kimpe, 2006 ▸; Maharramov *et al.*, 2011 ▸). Compounds incorporating thia­zolidine and azomethine structural motifs have also found applications in coordination chemistry, catalysis, crystal design and material science (Asadov *et al.*, 2016 ▸; Akbari Afkhami *et al.*, 2017 ▸; Maharramov *et al.*, 2018 ▸; Mahmudov *et al.*, 2019 ▸, 2020 ▸).
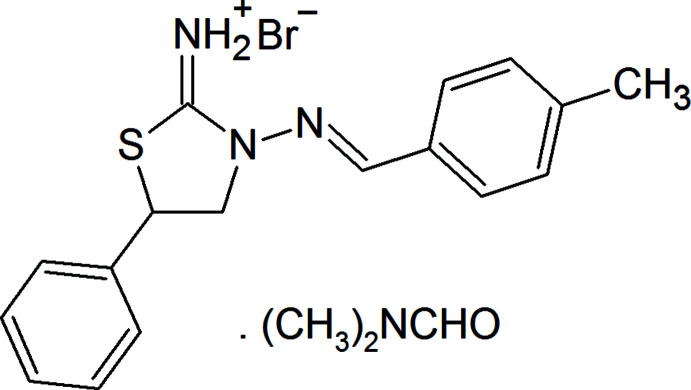


As part of our ongoing structural studies (Akkurt *et al.*, 2018*a* ▸,*b* ▸; Khalilov *et al.*, 2011 ▸, 2019 ▸), we report herein the crystal structure and Hirshfeld surface analysis of the title compound, (*E*)-3-[(4-methyl­benzyl­idene)amino]-5-phenylthiazolidin-2-iminium bromide *N*,*N*-di­methyl­formamide monosolvate.

## Structural commentary   

**Figure 1:**
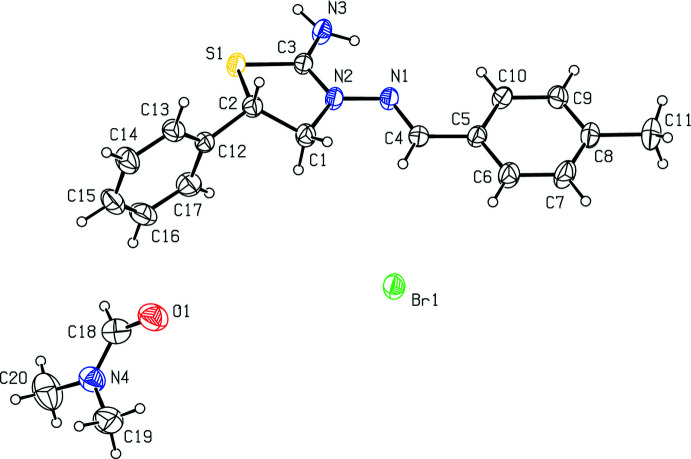
The mol­ecular structure of the title salt, showing displacement ellipsoids drawn at the 30% probability level.

As shown in Fig. 1 ▸, the central thia­zolidine ring (S1/N2/C1–C3) of the cation adopts an envelope conformation with puckering parameters (Cremer & Pople, 1975 ▸) *Q*(2) = 0.310 (3) Å and *φ*(2) = 42.2 (6)° with atom C2 as the flap. The C=N double bond [N1=C4 = 1.272 (4) Å] is in a Z configuration. The dihedral angle between the mean planes of the benzene (C5–C10) and phenyl (C12–C17) rings is 83.95 (18)° and they make dihedral angles of 16.60 (17) and 87.42 (17)°, respectively, with the mean plane of the thia­zolidine ring. The N2—N1—C4—C5 bridge that links the thia­zolidine and 4-methyl­benzene rings has a torsion angle of −176.8 (3)°.

## Supra­molecular features   

**Figure 2:**
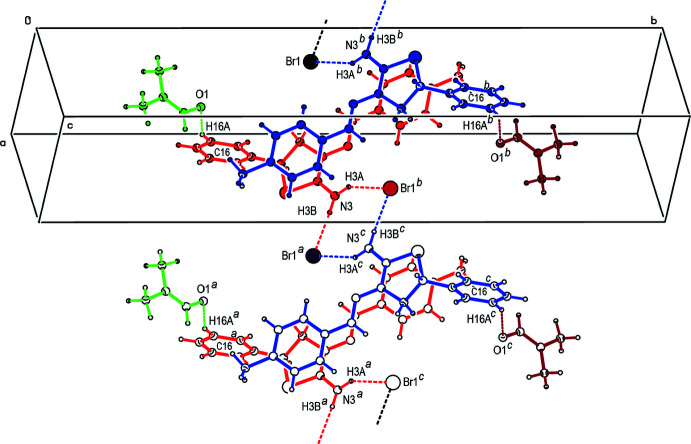
A view of hydrogen bonds between the cations, anions and *N*,*N*-di­methyl­formamide mol­ecules of the title salt. The N—H⋯Br hydrogen bonds and C—H⋯O contacts are shown as dashed lines. Symmetry codes: (*a*) 1 + *x*, *y*, 1 + *z*; (*b*) 1 − *x*, 1 − *y*, 1 − *z*; (*c*) 2 − *x*, 1 − *y*, 2 − *z*.

In the crystal, each cation is connected to two anions by N—H⋯Br hydrogen bonds forming an 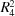
(8) motif parallel to the (10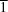) plane, while *N*,*N*-di­methyl­formamide mol­ecules are linked to the cations by C—H⋯O contacts (Table 1 ▸; Figs. 2 ▸, 3 ▸ and 4 ▸). Furthermore, van der Waals inter­actions between the cations, anions and *N*,*N*-di­methyl­formamide mol­ecules stabilize the crystal structure in three dimensions.

## Hirshfeld surface analysis   

**Figure 5:**
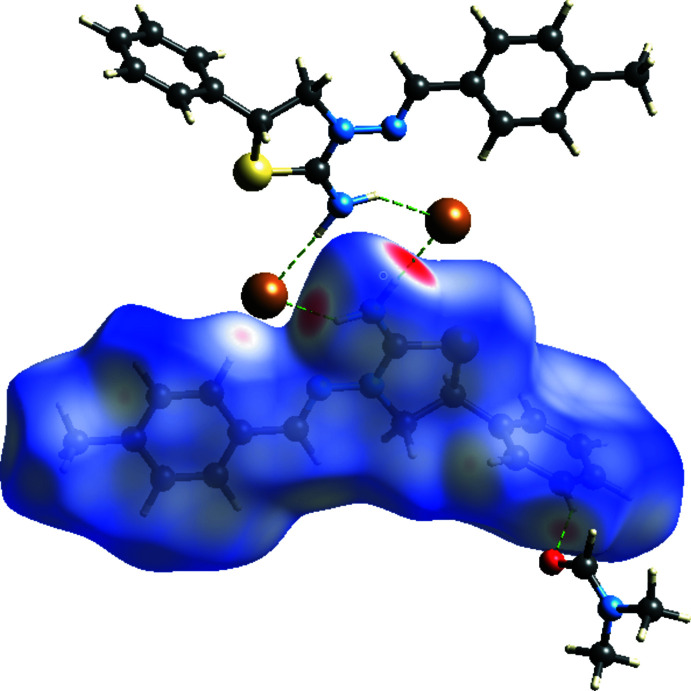
A view of the three-dimensional Hirshfeld surface for the title salt, plotted over *d*_norm_ in the range −0.4961 to 1.2178 a.u. N—H⋯Br hydrogen bonds and C—H⋯O contacts are shown.

**Figure 6:**
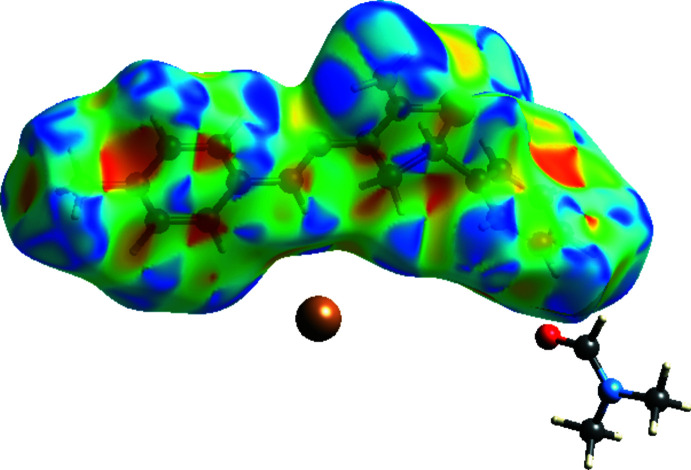
View of the three-dimensional Hirshfeld surface of the title salt plotted over shape-index.

Hirshfeld surface analysis (Spackman & Jayatilaka, 2009 ▸) was used to investigate the hydrogen bonds and inter­molecular inter­actions in the crystal structure. This was performed using *CrystalExplorer3.1* (Wolff *et al.*, 2012 ▸), and comprised *d*_norm_ surface plots and two-dimensional fingerprint plots (Spackman & McKinnon, 2002 ▸). The shorter and longer contacts are indicated as red and blue spots, respectively, on the Hirshfeld surfaces, and contacts with distances approximately equal to the sum of the van der Waals radii are represented as white spots. The contribution of inter­atomic contacts (Table 2 ▸) to the *d*_norm_ surface of the title compound is shown in Fig. 5 ▸. Fig. 6 ▸ indicates by the absence of red and blue triangles on the shape-index surface that π–π stacking inter­actions are not present in the crystal structure.

**Figure 7:**
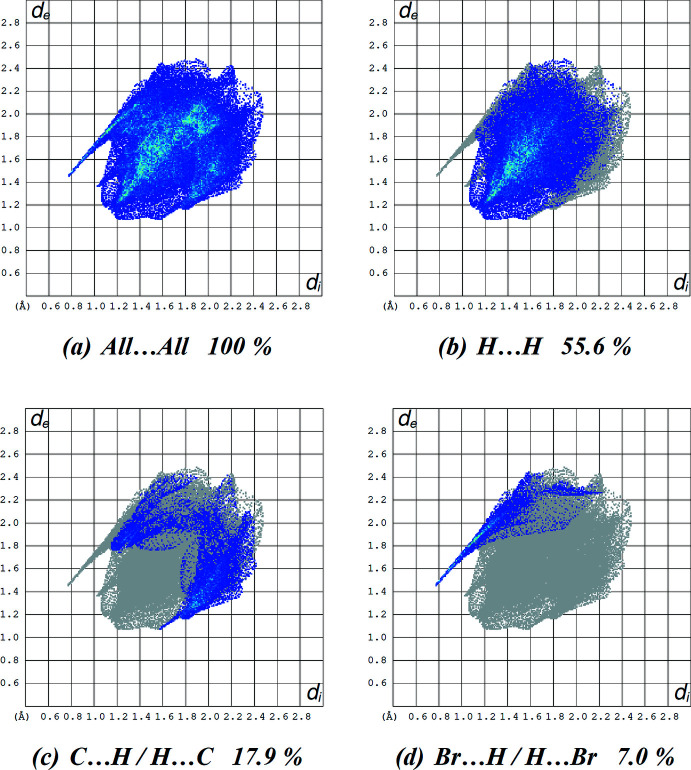
A view of the two-dimensional fingerprint plots for the title salt, showing (*a*) all inter­actions, and delineated into (*b*) H⋯H, (*c*) C⋯H/H⋯C and (*d*) Br⋯H/H⋯Br inter­actions. The *d*_i_ and *d*_e_ values are the closest inter­nal and external distances (in Å) from given points on the Hirshfeld surface.

Fig. 7 ▸(*a*) shows the 2D fingerprint plot of the sum of the contacts contributing to the Hirshfeld surface represented in normal mode while those delineated into H⋯H, C⋯H/H⋯C and Br⋯H/H⋯Br contacts are given in Fig. 7 ▸ *b–d*, respectively. The most significant inter­molecular inter­actions are the H⋯H inter­actions (55.6%) (Fig. 7 ▸ *b*). The reciprocal C⋯H/H⋯C inter­actions appear as two symmetrical broad wings with *d*_e_ + *d*_i_ ≃ 2.6 Å and contribute 17.9% to the Hirshfeld surface (Fig. 7 ▸ *c*). The reciprocal Br⋯H/H⋯Br inter­action with a 7.0% contribution is seen as branch of sharp symmetrical spikes at diagonal axes *d*_e_ + *d*_i_ ≃ 2.2 Å (Fig. 7 ▸ *d*). Furthermore, there are also O⋯H/H⋯O (3.2%), S⋯H/H⋯S (4.6%), N⋯C/C⋯N (3.8%), N⋯H/H⋯N (2.9%), S⋯C/C⋯S (2.4%), C⋯C (1.5%), Br⋯C/C⋯Br (0.2%), Br⋯S/S⋯Br (0.2%), N⋯N (0.4%) and N⋯S/S⋯N (0.5%) contacts (Table 3 ▸).

## Database survey   

A search of the Cambridge Structural Database CSD (Version 5.40, update of August 2019; Groom *et al.*, 2016 ▸) yielded eight hits for 2-thia­zolidiniminium compounds, with four of them reporting essentially the same cation [CSD refcodes WILBIC (Marthi *et al.*, 1994 ▸), WILBOI (Marthi *et al.*, 1994 ▸), WILBOI01 (Marthi *et al.*, 1994 ▸), YITCEJ (Martem’yanova *et al.*, 1993*a* ▸), YITCAF (Martem’yanova *et al.*, 1993*b* ▸) and YOPLUK (Marthi *et al.*, 1995 ▸)]. In all cases, the 3-N atom carries a C substituent, not N as found in the title compound. The first three crystal structures were determined for racemic (WILBIC; Marthi *et al.*, 1994 ▸) and two optically active samples (WILBOI and WILBOI01) of 3-(2-chloro-2-phenyl­eth­yl)-2-thia­zolidiniminium*p*-toluene­sulfonate. In all three structures, the most disordered fragment of these mol­ecules is the asymmetric C atom and the Cl atom attached to it. The disorder of the cation in the racemate corresponds to the presence of both enanti­omers at each site in the ratio 0.821 (3):0.179 (3). The system of hydrogen bonds connecting two cations and two anions into 12-membered rings is identical in the racemic and in the optically active crystals. YITCEJ (Martem’yanova *et al.*, 1993*a* ▸) is a product of the inter­action of 2-amino-5-methyl­thia­zoline with methyl iodide, with alkyl­ation at the endocylic N atom, while YITCAF (Martem’yanova *et al.*, 1993*b* ▸) is a product of the reaction of 3-nitro-5-meth­oxy-, 3-nitro-5-chloro- and 3-bromo-5-nitro­salicyl­aldehyde with the heterocyclic base to form the salt-like complexes.

The other closely related compounds are UDELUN (Akkurt *et al.*, 2018*a* ▸) and ZIJQAN (Akkurt *et al.*, 2018*b* ▸). In the crystal structure of UDELUN, the 3-N atom of the cation carries an N substituent, as found in the title compound. In the crystal, C—H⋯Br and N—H⋯Br hydrogen bonds link the components into a three-dimensional network with the cations and anions stacked along the *b*-axis direction. Weak C—H⋯π inter­actions and inversion-related Cl⋯Cl halogen bonds and C—Cl⋯π(ring) contacts also contribute to the mol­ecular packing. In the crystal of ZIJQAN, the cations, anions and water mol­ecules are linked into a three-dimensional network, which forms cross layers parallel to the (120) and (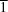
20) planes *via* O—H⋯Br, N—H⋯Br and N—H⋯N hydrogen bonds. Furthermore, C—H⋯π inter­actions also help in the stabilization of the mol­ecular packing.

Furthermore, in WILBIC, the thia­zolidine ring adopts a twist conformation. In one of two mol­ecules in the asymmetric unit of WILBOI, the thia­zolidine ring is essentially planar, in the other it adopts a twist conformation. In the two mol­ecules in the asymmetric unit of WILBOI01 and in YOPLUK, the thia­zolidine rings exhibit a twist conformation. In YITCAF, the disordered thia­zolidine ring has two components, which are planar. In YOPLUK, the thia­zolidine ring is slightly puckered, with the nitro­gen atom in an almost planar configuration. In the cations of UDELUN and ZIJQAN, the thia­zolidine rings have an envelope conformation.

## Synthesis and crystallization   

To a solution of 3-amino-5-phenyl­thia­zolidin-2-iminium bromide (1 mmol) in ethanol (20 ml) was added 4-methyl­benzaldehyde (1 mmol). The mixture was refluxed for 2 h and then cooled. The reaction product precipitated from the reaction mixture as colorless crystals, was collected by filtration, washed with cold acetone (yield 54%; m.p. 501–502 K), and recrystallized from di­methyl­formamide to obtain single crystals.

^1^H NMR (300 MHz, DMSO-*d*_6_) : 2.33 (*s*, 3H, CH_3_); 4.55 (*k*, 1H, CH_2_, ^3^ *J*_H–H_ = 6.6); 4,88 (*t*, 1H, CH_2_, ^3^ *J*_H–H_ = 8.1); 5.60 (*t*, 1H, CH—Ar, ^3^ *J*_H–H_ = 7.5); 7.28–7.98 (*m*, 9H, 9Ar—H); 8.41 (*s*, 1H, CH=); 10.33 (*s*, 2H, N^+^H=). ^13^C NMR (75 MHz, DMSO-*d*_6_): 21.27; 45.36; 55.90; 127.79; 128.69; 128.86; 129.09; 129.46; 130.21; 137.50; 141.68; 151.04; 167.50. MS (ESI), *m*/*z*: 296.40 [C_17_H_18_N_3_S]^+^ and 79.88 Br^−^.

## Refinement   

Crystal data, data collection and structure refinement details are summarized in Table 4 ▸. All H atoms were placed geometrically (N—H = 0.90 Å and C—H = 0.93–0.98 Å) and refined as riding atoms with *U*_iso_(H) = 1.2 or 1.5*U*_eq_(C, N).

## Supplementary Material

Crystal structure: contains datablock(s) I. DOI: 10.1107/S2056989020012712/jy2001sup1.cif

Structure factors: contains datablock(s) I. DOI: 10.1107/S2056989020012712/jy2001Isup2.hkl

CCDC reference: 1837125

Additional supporting information:  crystallographic information; 3D view; checkCIF report

## Figures and Tables

**Figure 3:**
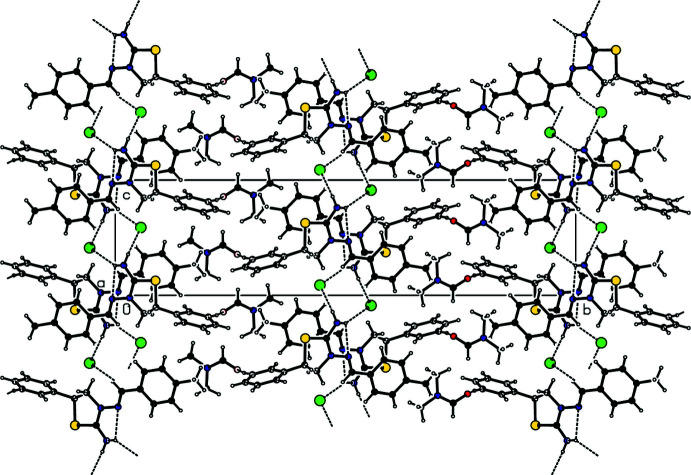
Crystal packing for the title salt viewed along the *a*-axis direction. Dashed lines indicate N—H⋯Br hydrogen bonds and C—H⋯O contacts.

**Figure 4:**
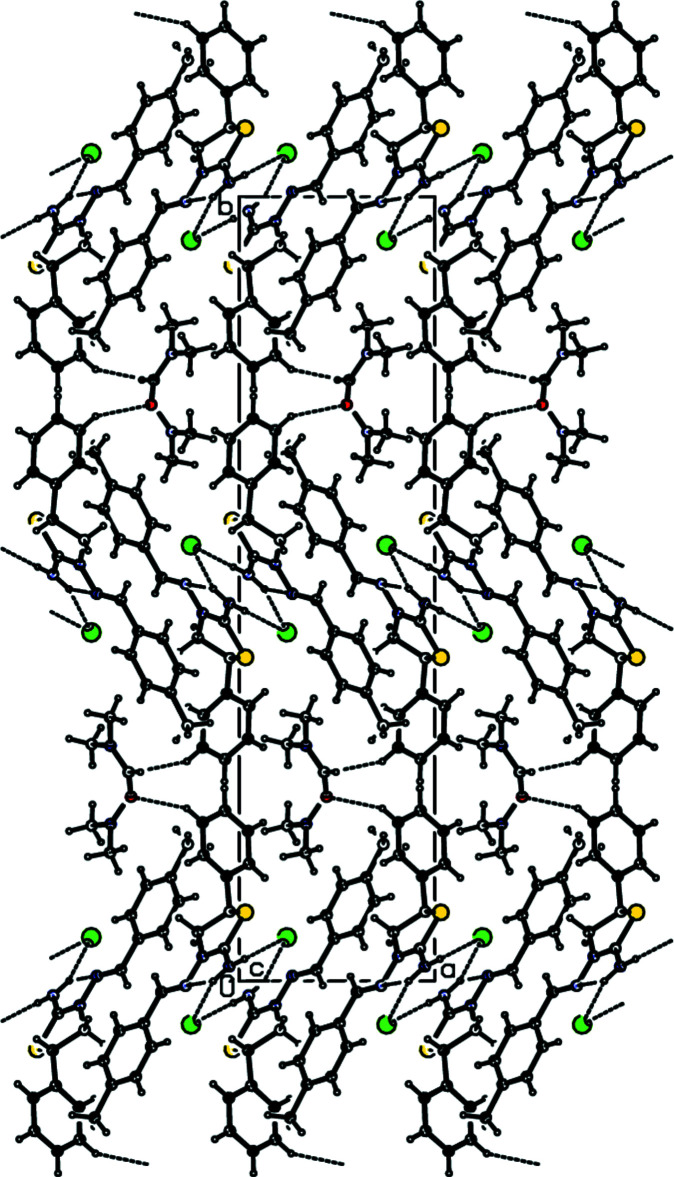
Crystal packing of the title salt viewed along the *c*-axis direction. Dashed lines indicate N—H⋯Br and C—H⋯O contacts.

**Table 1:** Hydrogen-bond geometry (Å, °)

*D*—H⋯*A*	*D*—H	H⋯*A*	*D*⋯*A*	*D*—H⋯*A*
N3—H3*A*⋯Br1^i^	0.90	2.57	3.368 (3)	148
N3—H3*B*⋯Br1^ii^	0.90	2.35	3.243 (2)	175
C16—H16*A*⋯O1	0.93	2.54	3.391 (6)	153

Symmetry codes: (i) 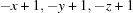; (ii) 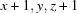.

**Table 2:** Summary of short inter­atomic contacts (Å) in the title salt

Contact	Distance	Symmetry operation
H3*B*⋯Br1	2.35	1 + *x*, *y*, 1 + *z*
N1⋯S1	3.533 (3)	2 − *x*, 1 − *y*, 1 − *z*
H3*A*⋯Br1	2.57	1 − *x*, 1 − *y*, 1 − *z*
C9⋯H19*C*	2.78	1 − *x*, 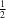 + *y*, 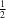 − *z*
H17*A*⋯H9*A*	2.47	1 − *x*, 1 − *y*, 1 − *z*
H6*A*⋯H1*A*	2.50	1 − *x*, 1 − *y*, −*z*
H16*A*⋯O1	2.54	*x*, *y*, *z*
H16*A*⋯H18*A*	2.55	*x*, 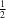 − *y*, − 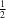 + *z*
H4*A*⋯Br1	3.06	*x*, *y*, *z*
H13*A*⋯Br1	3.08	1 + *x*, *y*, *z*
H14*A*⋯O1	2.84	1 + *x*, *y*, *z*
O1⋯H18*A*	2.76	*x*, 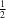 − *y*, − 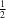 + *z*
C20⋯Br1	3.736 (5)	*x*, 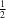 − *y*, 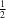 + *z*

**Table 3:** Percentage contributions of inter­atomic contacts to the Hirshfeld surface for the title salt

Contact	Percentage contribution
H⋯H	55.6
C⋯H/H⋯C	17.9
Br⋯H/H⋯Br	7.0
S⋯H/H⋯S	4.6
N⋯C/C⋯N	3.8
O⋯H/H⋯O	3.2
N⋯H/H⋯N	2.9
S⋯C/C⋯S	2.4
C⋯C	1.5
N⋯S/S⋯N	0.5
N⋯N	0.4
Br⋯C/C⋯Br	0.2
Br⋯S/S⋯Br	0.2

**Table 4:** Experimental details

Crystal data	
Chemical formula	C_17_H_18_N_3_S^+^·Br^−^·C_3_H_7_NO
*M*_r_	449.41
Crystal system, space group	Monoclinic, *P*2_1_/*c*
Temperature (K)	296
*a*, *b*, *c* (Å)	8.4326 (6), 31.778 (2), 8.4680 (6)
β (°)	110.052 (2)
*V* (Å^3^)	2131.6 (3)
*Z*	4
Radiation type	Mo *K*α
μ (mm^−1^)	2.04
Crystal size (mm)	0.18 × 0.14 × 0.10

Data collection	
Diffractometer	Bruker APEXII CCD
Absorption correction	Multi-scan (*SADABS*; Bruker, 2003 ▸)
*T*_min_, *T*_max_	0.702, 0.807
No. of measured, independent and observed [*I* > 2σ(*I*)] reflections	29638, 4039, 2873
*R*_int_	0.076
(sin θ/λ)_max_ (Å^−1^)	0.609

Refinement	
*R*[*F* ^2^ > 2σ(*F* ^2^)], *wR*(*F* ^2^), *S*	0.038, 0.087, 1.03
No. of reflections	4039
No. of parameters	248
H-atom treatment	H-atom parameters constrained
Δρ_max_, Δρ_min_ (e Å^−3^)	0.38, −0.61

Computer programs: *APEX2* and *SAINT* (Bruker, 2003 ▸), *SHELXT2014/5* (Sheldrick, 2015*a* ▸), *SHELXL2018/3* (Sheldrick, 2015*b* ▸), *ORTEP-3 for Windows* (Farrugia, 2012 ▸) and *PLATON* (Spek, 2020 ▸).

## supplementary crystallographic information

### Crystal data

**Table:**

C_17_H_18_N_3_S^+^·Br^−^·C_3_H_7_NO	*F*(000) = 928
*M**_r_* = 449.41	*D*_x_ = 1.400 Mg m^−^^3^
Monoclinic, *P*2_1_/*c*	Mo *K*α radiation, λ = 0.71073 Å
*a* = 8.4326 (6) Å	Cell parameters from 7783 reflections
*b* = 31.778 (2) Å	θ = 2.6–25.5°
*c* = 8.4680 (6) Å	µ = 2.04 mm^−^^1^
β = 110.052 (2)°	*T* = 296 K
*V* = 2131.6 (3) Å^3^	Plate, colourless
*Z* = 4	0.18 × 0.14 × 0.10 mm

### Data collection

**Table:**

Bruker APEXII CCD diffractometer	2873 reflections with *I* > 2σ(*I*)
φ and ω scans	*R*_int_ = 0.076
Absorption correction: multi-scan (SADABS; Bruker, 2003)	θ_max_ = 25.7°, θ_min_ = 2.6°
*T*_min_ = 0.702, *T*_max_ = 0.807	*h* = −10→10
29638 measured reflections	*k* = −38→38
4039 independent reflections	*l* = −10→10

### Refinement

**Table:**

Refinement on *F*^2^	Hydrogen site location: mixed
Least-squares matrix: full	H-atom parameters constrained
*R*[*F*^2^ > 2σ(*F*^2^)] = 0.038	*w* = 1/[σ^2^(*F*_o_^2^) + (0.0236*P*)^2^ + 1.7903*P*] where *P* = (*F*_o_^2^ + 2*F*_c_^2^)/3
*wR*(*F*^2^) = 0.087	(Δ/σ)_max_ = 0.001
*S* = 1.03	Δρ_max_ = 0.43 e Å^−^^3^
4039 reflections	Δρ_min_ = −0.53 e Å^−^^3^
248 parameters	Extinction correction: *SHELXL2018/3* (Sheldrick, 2015b), Fc^*^=kFc[1+0.001xFc^2^λ^3^/sin(2θ)]^-1/4^
0 restraints	Extinction coefficient: 0.0081 (7)

### Special details

**Table:**

Geometry. All esds (except the esd in the dihedral angle between two l.s. planes) are estimated using the full covariance matrix. The cell esds are taken into account individually in the estimation of esds in distances, angles and torsion angles; correlations between esds in cell parameters are only used when they are defined by crystal symmetry. An approximate (isotropic) treatment of cell esds is used for estimating esds involving l.s. planes.

### Fractional atomic coordinates and isotropic or equivalent isotropic displacement parameters (Å^2^)

**Table:**

	*x*	*y*	*z*	*U*_iso_*/*U*_eq_	
Br1	0.24500 (5)	0.44440 (2)	0.08820 (4)	0.06338 (16)	
N1	0.7272 (3)	0.50622 (7)	0.4672 (3)	0.0454 (6)	
N2	0.8146 (3)	0.46878 (7)	0.4775 (3)	0.0462 (6)	
N3	0.9508 (3)	0.48154 (8)	0.7609 (3)	0.0542 (7)	
H3A	0.869458	0.500223	0.757513	0.065*	
H3B	1.029166	0.472122	0.855943	0.065*	
N4	0.3431 (4)	0.20008 (10)	0.3700 (4)	0.0690 (8)	
O1	0.4488 (4)	0.26513 (10)	0.3557 (4)	0.0891 (8)	
S1	1.03303 (10)	0.41267 (2)	0.62732 (10)	0.0505 (2)	
C1	0.7900 (4)	0.43751 (10)	0.3435 (4)	0.0535 (8)	
H1A	0.771425	0.451273	0.236395	0.064*	
H1B	0.693711	0.419711	0.333814	0.064*	
C2	0.9532 (4)	0.41145 (9)	0.3953 (4)	0.0478 (7)	
H2A	1.034938	0.425774	0.354929	0.057*	
C3	0.9255 (4)	0.45875 (9)	0.6271 (4)	0.0429 (7)	
C4	0.6040 (4)	0.51408 (9)	0.3334 (4)	0.0487 (7)	
H4A	0.571043	0.494228	0.247559	0.058*	
C5	0.5142 (4)	0.55408 (9)	0.3137 (4)	0.0459 (7)	
C6	0.3893 (4)	0.56328 (11)	0.1627 (4)	0.0628 (9)	
H6A	0.359656	0.543477	0.076567	0.075*	
C7	0.3078 (5)	0.60187 (12)	0.1387 (5)	0.0695 (10)	
H7A	0.223309	0.607480	0.036564	0.083*	
C8	0.3491 (4)	0.63205 (10)	0.2626 (4)	0.0532 (8)	
C9	0.4726 (4)	0.62221 (10)	0.4137 (4)	0.0521 (8)	
H9A	0.502433	0.642090	0.499513	0.063*	
C10	0.5529 (4)	0.58380 (10)	0.4409 (4)	0.0485 (7)	
H10A	0.633347	0.577780	0.545053	0.058*	
C11	0.2619 (5)	0.67440 (11)	0.2337 (5)	0.0770 (11)	
H11A	0.261055	0.685750	0.128290	0.116*	
H11B	0.321407	0.693239	0.322952	0.116*	
H11C	0.147992	0.671094	0.231326	0.116*	
C12	0.9372 (4)	0.36634 (9)	0.3360 (4)	0.0454 (7)	
C13	1.0517 (4)	0.35074 (10)	0.2659 (4)	0.0565 (8)	
H13A	1.132036	0.368508	0.248893	0.068*	
C14	1.0464 (5)	0.30867 (11)	0.2212 (5)	0.0709 (10)	
H14A	1.123460	0.298109	0.174733	0.085*	
C15	0.9283 (5)	0.28289 (11)	0.2456 (5)	0.0734 (11)	
H15A	0.925525	0.254635	0.216122	0.088*	
C16	0.8144 (5)	0.29779 (12)	0.3122 (5)	0.0732 (11)	
H16A	0.733331	0.279913	0.326952	0.088*	
C17	0.8191 (4)	0.33936 (11)	0.3578 (5)	0.0629 (9)	
H17A	0.741175	0.349390	0.404218	0.076*	
C18	0.4359 (5)	0.23379 (14)	0.4343 (5)	0.0757 (11)	
H18A	0.496399	0.233544	0.549077	0.091*	
C19	0.2468 (6)	0.19812 (15)	0.1924 (6)	0.0934 (13)	
H19A	0.250819	0.224946	0.141811	0.140*	
H19B	0.131711	0.191116	0.176912	0.140*	
H19C	0.293996	0.176990	0.140581	0.140*	
C20	0.3410 (7)	0.16367 (15)	0.4722 (7)	0.1129 (17)	
H20A	0.410879	0.168951	0.586481	0.169*	
H20B	0.383236	0.139639	0.430398	0.169*	
H20C	0.227360	0.158275	0.467287	0.169*	

### Atomic displacement parameters (Å^2^)

**Table:**

	*U*^11^	*U*^22^	*U*^33^	*U*^12^	*U*^13^	*U*^23^
Br1	0.0746 (3)	0.0552 (2)	0.0497 (2)	0.01350 (18)	0.00753 (16)	−0.00653 (16)
N1	0.0476 (14)	0.0418 (14)	0.0449 (14)	0.0098 (11)	0.0133 (12)	0.0017 (11)
N2	0.0505 (14)	0.0416 (14)	0.0405 (13)	0.0138 (12)	0.0077 (11)	−0.0006 (11)
N3	0.0609 (17)	0.0538 (16)	0.0409 (14)	0.0202 (13)	0.0086 (12)	0.0019 (12)
N4	0.069 (2)	0.065 (2)	0.078 (2)	−0.0069 (16)	0.0321 (17)	−0.0073 (17)
O1	0.086 (2)	0.079 (2)	0.104 (2)	−0.0168 (16)	0.0357 (17)	−0.0018 (18)
S1	0.0531 (5)	0.0452 (4)	0.0473 (4)	0.0140 (4)	0.0095 (3)	0.0008 (4)
C1	0.0604 (19)	0.0461 (19)	0.0471 (18)	0.0104 (15)	0.0094 (15)	−0.0076 (14)
C2	0.0498 (18)	0.0444 (17)	0.0476 (17)	0.0037 (14)	0.0147 (14)	−0.0016 (14)
C3	0.0442 (16)	0.0406 (16)	0.0413 (17)	0.0064 (13)	0.0113 (13)	0.0000 (13)
C4	0.0479 (18)	0.0434 (17)	0.0497 (18)	0.0040 (14)	0.0102 (15)	−0.0016 (14)
C5	0.0430 (16)	0.0447 (17)	0.0470 (17)	0.0054 (14)	0.0115 (13)	0.0078 (14)
C6	0.072 (2)	0.056 (2)	0.0482 (19)	0.0137 (18)	0.0048 (16)	0.0026 (16)
C7	0.070 (2)	0.072 (2)	0.054 (2)	0.023 (2)	0.0047 (17)	0.0182 (19)
C8	0.0527 (19)	0.0461 (18)	0.064 (2)	0.0116 (15)	0.0239 (16)	0.0149 (16)
C9	0.0494 (18)	0.0470 (19)	0.062 (2)	0.0054 (15)	0.0219 (16)	−0.0039 (15)
C10	0.0431 (17)	0.0530 (19)	0.0463 (17)	0.0099 (15)	0.0112 (13)	0.0016 (15)
C11	0.084 (3)	0.058 (2)	0.091 (3)	0.028 (2)	0.032 (2)	0.022 (2)
C12	0.0468 (17)	0.0408 (16)	0.0432 (17)	0.0035 (14)	0.0086 (13)	−0.0027 (13)
C13	0.0511 (19)	0.050 (2)	0.071 (2)	−0.0044 (16)	0.0246 (17)	−0.0015 (17)
C14	0.072 (2)	0.054 (2)	0.096 (3)	0.0031 (19)	0.042 (2)	−0.016 (2)
C15	0.082 (3)	0.044 (2)	0.094 (3)	−0.0072 (19)	0.030 (2)	−0.0156 (19)
C16	0.068 (2)	0.060 (2)	0.090 (3)	−0.021 (2)	0.025 (2)	−0.010 (2)
C17	0.053 (2)	0.065 (2)	0.077 (2)	−0.0029 (18)	0.0293 (18)	−0.0042 (19)
C18	0.068 (2)	0.086 (3)	0.073 (3)	−0.008 (2)	0.023 (2)	−0.007 (2)
C19	0.089 (3)	0.090 (3)	0.092 (3)	−0.002 (3)	0.018 (2)	−0.026 (3)
C20	0.142 (5)	0.082 (3)	0.139 (5)	−0.005 (3)	0.079 (4)	0.021 (3)

### Geometric parameters (Å, º)

**Table:**

N1—C4	1.272 (4)	C8—C9	1.380 (4)
N1—N2	1.386 (3)	C8—C11	1.513 (4)
N2—C3	1.330 (4)	C9—C10	1.376 (4)
N2—C1	1.468 (4)	C9—H9A	0.9300
N3—C3	1.299 (4)	C10—H10A	0.9300
N3—H3A	0.9000	C11—H11A	0.9600
N3—H3B	0.9000	C11—H11B	0.9600
N4—C18	1.328 (5)	C11—H11C	0.9600
N4—C19	1.445 (5)	C12—C17	1.374 (4)
N4—C20	1.448 (5)	C12—C13	1.387 (4)
O1—C18	1.223 (5)	C13—C14	1.386 (5)
S1—C3	1.722 (3)	C13—H13A	0.9300
S1—C2	1.846 (3)	C14—C15	1.359 (5)
C1—C2	1.535 (4)	C14—H14A	0.9300
C1—H1A	0.9700	C15—C16	1.355 (5)
C1—H1B	0.9700	C15—H15A	0.9300
C2—C12	1.510 (4)	C16—C17	1.373 (5)
C2—H2A	0.9800	C16—H16A	0.9300
C4—C5	1.459 (4)	C17—H17A	0.9300
C4—H4A	0.9300	C18—H18A	0.9300
C5—C6	1.381 (4)	C19—H19A	0.9600
C5—C10	1.385 (4)	C19—H19B	0.9600
C6—C7	1.386 (5)	C19—H19C	0.9600
C6—H6A	0.9300	C20—H20A	0.9600
C7—C8	1.375 (5)	C20—H20B	0.9600
C7—H7A	0.9300	C20—H20C	0.9600
			
C4—N1—N2	118.6 (2)	C8—C9—H9A	119.1
C3—N2—N1	116.8 (2)	C9—C10—C5	120.3 (3)
C3—N2—C1	116.1 (2)	C9—C10—H10A	119.9
N1—N2—C1	127.0 (2)	C5—C10—H10A	119.9
C3—N3—H3A	116.4	C8—C11—H11A	109.5
C3—N3—H3B	116.4	C8—C11—H11B	109.5
H3A—N3—H3B	124.4	H11A—C11—H11B	109.5
C18—N4—C19	120.2 (4)	C8—C11—H11C	109.5
C18—N4—C20	121.7 (4)	H11A—C11—H11C	109.5
C19—N4—C20	118.0 (4)	H11B—C11—H11C	109.5
C3—S1—C2	90.96 (13)	C17—C12—C13	118.5 (3)
N2—C1—C2	105.6 (2)	C17—C12—C2	122.3 (3)
N2—C1—H1A	110.6	C13—C12—C2	119.1 (3)
C2—C1—H1A	110.6	C14—C13—C12	120.0 (3)
N2—C1—H1B	110.6	C14—C13—H13A	120.0
C2—C1—H1B	110.6	C12—C13—H13A	120.0
H1A—C1—H1B	108.7	C15—C14—C13	119.8 (3)
C12—C2—C1	116.6 (3)	C15—C14—H14A	120.1
C12—C2—S1	109.4 (2)	C13—C14—H14A	120.1
C1—C2—S1	104.9 (2)	C16—C15—C14	120.9 (3)
C12—C2—H2A	108.6	C16—C15—H15A	119.6
C1—C2—H2A	108.6	C14—C15—H15A	119.6
S1—C2—H2A	108.6	C15—C16—C17	119.8 (3)
N3—C3—N2	123.2 (3)	C15—C16—H16A	120.1
N3—C3—S1	123.0 (2)	C17—C16—H16A	120.1
N2—C3—S1	113.8 (2)	C16—C17—C12	121.0 (3)
N1—C4—C5	120.4 (3)	C16—C17—H17A	119.5
N1—C4—H4A	119.8	C12—C17—H17A	119.5
C5—C4—H4A	119.8	O1—C18—N4	125.7 (4)
C6—C5—C10	118.5 (3)	O1—C18—H18A	117.2
C6—C5—C4	119.4 (3)	N4—C18—H18A	117.2
C10—C5—C4	122.0 (3)	N4—C19—H19A	109.5
C5—C6—C7	120.4 (3)	N4—C19—H19B	109.5
C5—C6—H6A	119.8	H19A—C19—H19B	109.5
C7—C6—H6A	119.8	N4—C19—H19C	109.5
C8—C7—C6	121.5 (3)	H19A—C19—H19C	109.5
C8—C7—H7A	119.3	H19B—C19—H19C	109.5
C6—C7—H7A	119.3	N4—C20—H20A	109.5
C7—C8—C9	117.6 (3)	N4—C20—H20B	109.5
C7—C8—C11	121.0 (3)	H20A—C20—H20B	109.5
C9—C8—C11	121.5 (3)	N4—C20—H20C	109.5
C10—C9—C8	121.8 (3)	H20A—C20—H20C	109.5
C10—C9—H9A	119.1	H20B—C20—H20C	109.5
			
C4—N1—N2—C3	−170.3 (3)	C6—C7—C8—C11	178.7 (4)
C4—N1—N2—C1	5.3 (4)	C7—C8—C9—C10	0.2 (5)
C3—N2—C1—C2	−24.2 (4)	C11—C8—C9—C10	−179.8 (3)
N1—N2—C1—C2	160.2 (3)	C8—C9—C10—C5	1.8 (5)
N2—C1—C2—C12	150.8 (3)	C6—C5—C10—C9	−2.6 (5)
N2—C1—C2—S1	29.7 (3)	C4—C5—C10—C9	175.9 (3)
C3—S1—C2—C12	−149.7 (2)	C1—C2—C12—C17	−50.1 (4)
C3—S1—C2—C1	−23.9 (2)	S1—C2—C12—C17	68.6 (3)
N1—N2—C3—N3	1.9 (4)	C1—C2—C12—C13	133.7 (3)
C1—N2—C3—N3	−174.2 (3)	S1—C2—C12—C13	−107.5 (3)
N1—N2—C3—S1	−178.1 (2)	C17—C12—C13—C14	−0.7 (5)
C1—N2—C3—S1	5.8 (4)	C2—C12—C13—C14	175.6 (3)
C2—S1—C3—N3	−168.3 (3)	C12—C13—C14—C15	0.3 (6)
C2—S1—C3—N2	11.6 (2)	C13—C14—C15—C16	0.4 (6)
N2—N1—C4—C5	−176.8 (3)	C14—C15—C16—C17	−0.8 (6)
N1—C4—C5—C6	175.2 (3)	C15—C16—C17—C12	0.4 (6)
N1—C4—C5—C10	−3.3 (5)	C13—C12—C17—C16	0.3 (5)
C10—C5—C6—C7	1.5 (5)	C2—C12—C17—C16	−175.9 (3)
C4—C5—C6—C7	−177.0 (3)	C19—N4—C18—O1	0.5 (6)
C5—C6—C7—C8	0.5 (6)	C20—N4—C18—O1	177.5 (4)
C6—C7—C8—C9	−1.4 (5)		

### Hydrogen-bond geometry (Å, º)

**Table:**

*D*—H···*A*	*D*—H	H···*A*	*D*···*A*	*D*—H···*A*
N3—H3*A*···Br1^i^	0.90	2.57	3.368 (3)	148
N3—H3*B*···Br1^ii^	0.90	2.35	3.243 (2)	175
C16—H16*A*···O1	0.93	2.54	3.391 (6)	153

Symmetry codes: (i) −*x*+1, −*y*+1, −*z*+1; (ii) *x*+1, *y*, *z*+1.
